# The prognostic utility of bedside assessment of adults hospitalized with malaria in Myanmar: a retrospective analysis

**DOI:** 10.1186/s12936-015-0549-y

**Published:** 2015-02-07

**Authors:** Myat Kaung, Tint Tint Kyi, Ne Myo Aung, Myat Phone Kyaw, Myo Min, Zaw Win Htet, Nicholas M Anstey, Mar Mar Kyi, Josh Hanson

**Affiliations:** Hpa-an Hospital, Hpa-an, Kayin State Myanmar; Insein Hospital, Insein Township, Yangon, Myanmar; Department of Medical Research (Lower Myanmar), Yangon, Myanmar; Myanmar Medical Association, Yangon, Myanmar; Menzies School of Health Research, Charles Darwin University, Darwin, Australia

**Keywords:** Severe malaria, Clinical prediction tools, Myanmar, Pulse oximetry, Vivax malaria

## Abstract

**Background:**

Data collected in clinical trials have been used to develop scoring systems that identify adults with malaria at greatest risk of death. One of these, the RCAM score, can be simply determined by measuring a patient’s Glasgow Coma Score and respiratory rate on admission to hospital. However the safety of using the RCAM score to define high-risk patients has not been assessed outside of the clinical trial setting.

**Methods:**

A retrospective audit of medical records of all adults admitted with a diagnosis of malaria to two tertiary referral hospitals in Lower Myanmar in 2013 was undertaken. An RCAM score was calculated in all patients and related to their subsequent clinical course.

**Results:**

The recent decline in malaria hospitalizations at both sites continued in 2013. During the year 90 adults were hospitalized with malaria; 62 (69%) had *Plasmodium falciparum* mono-infection, 11 (12%) had *Plasmodium vivax* mono-infection, 17 (19%) had mixed infection. All seven (7.7%) deaths occurred in patients infected with *P. falciparum*. An admission RCAM score <2 identified all the patients that would survive to discharge (positive predictive value (95% confidence interval (CI)) 100% (94.9-100%) and also predicted a requirement for less supportive care: 9/70 (13%) patients with an admission RCAM score <2 required supportive care (blood transfusion, vasopressor support or oxygen supplementation) during their hospitalization compared with 12/20 (60%) patients with an admission RCAM score ≥2 (p < 0.0001). No patient with *P. vivax* mono-infection required supportive care during their hospitalization. Patients with an oxygen saturation ≤95% on room air on admission were more likely to die before discharge (odds ratio 17.3 (95% CI: 2.9-101.2) than patients with a higher oxygen saturation (p = 0.002).

**Conclusions:**

Even outside a clinical trial setting the RCAM score reliably identifies adults with malaria who are at greatest risk of death and can be safely used in the initial triage and management of these patients.

## Background

In an effort to reduce malaria-related mortality, the World Health Organization (WHO) has proposed criteria to define severe malaria, recommending that patients meeting these criteria receive prompt parenteral anti-malarial therapy and supportive treatment on an acute medical ward [1]. Earlier iterations of this definition [2,3] had limited practical utility: the sheer number of criteria (16 in the 2012 handbook) made its everyday use challenging. In addition strongly prognostic indices (metabolic acidosis) were listed alongside indices of limited prognostic value (prostration); manifestations appearing predominantly in adults (pulmonary oedema, renal impairment) were recorded next to those relevant mainly to the paediatric population (severe anaemia); and some criteria were relatively uncommon (haemoglobinuria and abnormal spontaneous bleeding). Perhaps most importantly however, many of the criteria required laboratory and radiology services that are rarely available in a resource-limited setting where patients with severe malaria are almost universally managed [4]. Recognizing these shortcomings, the most recent WHO consensus statement on severe malaria has placed a greater emphasis on clinical assessment in an effort to provide more practical guidance for health workers [1]. It is suggested that in a resource-limited setting patient management can be guided – at least initially – by findings at the bedside.

It has repeatedly been shown that the degree of metabolic acidosis in patients with malaria is strongly predictive of a complicated course and death [5-8]. As the physiological response to this metabolic acidosis is hyperventilation [9], the respiratory rate can be used as a crude bedside measure of acidosis [8]. The other main determinant of outcome with malaria is the level of consciousness [8,10,11], measured with the Glasgow Coma Score (GCS). The GCS and the respiratory rate are combined in adults to calculate the RCAM score, a clinical prediction tool that can be calculated at the bedside by even inexperienced health workers in less than 60 seconds (Table 1) [8]. When applied retrospectively to patients enrolled in studies of adults with malaria the RCAM score calculated at admission helped identify the patients that were more likely to survive [8,11], potentially facilitating their triage and the distribution of limited health resources [12].

**Table 1:** **Calculation of the RCAM score**

	**Score**		
**Variable**	**0 (normal)**	**1 (abnormal)**	**2 (very abnormal)**
GCS	15	11 to 14 (inclusive)	≤10
Respiratory rate	<20	20 to 39 (inclusive)	≥40

RCAM score (0–4) is calculated as the respiratory rate score (0–2) plus the GCS score (0–2).

The clinical trials that generated data for previous studies assessing the RCAM score were all performed in poorly resourced hospitals; however the patients in these studies were not receiving usual care. Some studies occurred in a specialized research ward [13], others used point of care testing which allowed clinicians more timely access to a broad range of pathology results [14]; almost all the studies had dedicated study clinicians and financial resources that would not be accessible outside of the trial setting. To determine if the RCAM score might be a reliable tool in the everyday management of adults with malaria in a resource-limited setting, a retrospective audit of all adult cases of malaria admitted to two referral hospitals in Myanmar was performed.

## Methods

### Study sites

The audit was comprised of adults admitted to Hpa-an Hospital and Insein General Hospital with a diagnosis of malaria between 1 January and 31 December, 2013. Hpa-an Hospital is the largest hospital in Kayin state (population 1.57 million), a state that neighbours Thailand to the east. Insein General Hospital is one of seven major public general hospitals in the Yangon region (population 5.6 million). The two hospitals serve populations in which there is low transmission of malaria. Cases occur year round although there is a peak in transmission during the mid-year wet season. At Hpa-an Hospital patients are generally socio-economically disadvantaged and live predominantly in surrounding rural villages; Insein General Hospital serves a predominantly urban population, which is also socio-economically disadvantaged.

### Limited resource setting

Laboratory services are limited at both sites; accordingly many laboratory tests are not routinely collected. Basic biochemistry (including plasma electrolytes, blood urea nitrogen, plasma creatinine, and liver function tests) can be measured and a full blood examination performed, however the laboratory services in both hospitals are only routinely available between 08.00 and 16.00 hours and six hours usually elapse before results are available. Private laboratories can provide out of hours results, however the extra cost of this service precludes its use by the vast majority of patients at both sites. Even if the patients have sufficient funds to access the private system, as specimens must be transported off site, results are rarely available within six hours. Plasma lactate cannot be measured at either hospital or in local private laboratories. Thick and thin blood films for malaria can be performed at any time of day, but as there is a turnaround time of several hours, clinicians usually rely on rapid diagnostic tests (RDTs) to make the diagnosis if the clinical suspicion is high.

Other diagnostic services and supportive care are also limited. Both hospitals have a radiology department where plain X-rays can be performed on a stable, portable patient; however neither site can perform mobile X-rays. Both sites lack facilities for renal replacement therapy (RRT) and neither have a functioning intensive care unit (ICU). Patients in Insein Hospital who require RRT must be transferred 25 km (one hour by road) although it is not logistically possible to transfer an acutely critically ill patient. Patients admitted to Hpa-an Hospital are at least five hours by motorway from the nearest unit providing RRT, which means that delivery of this therapy to the acutely ill patient is effectively impossible.

### Ethical approval

Ethical approval was granted for this audit by the Myanmar Ministry of Health and by the Menzies School of Health Research.

### Data collection

Data were retrospectively collected from medical records. Malaria was diagnosed with immunochromatographic RDTs (SD Bioline Malaria Ag P.f/P.v (05FK80), Standard Diagnostics, Republic of Korea). The malaria diagnosis and species was confirmed using semi-quantitative thick and thin films read by experienced technicians in the hospital laboratories. Demographic, clinical and laboratory data were entered into a standardized data collection form and entered into an anonymous database. Severity criteria from the 2012 WHO practical handbook for the management of severe malaria [2] were specifically sought in the medical record.

### Statistical analysis

Statistical analysis was performed using statistical software (Stata 10.0, Statacorp, Texas USA). Groups were analysed using the Kruskal-Wallis and Chi-squared tests.

## Results

**Figure 1:**
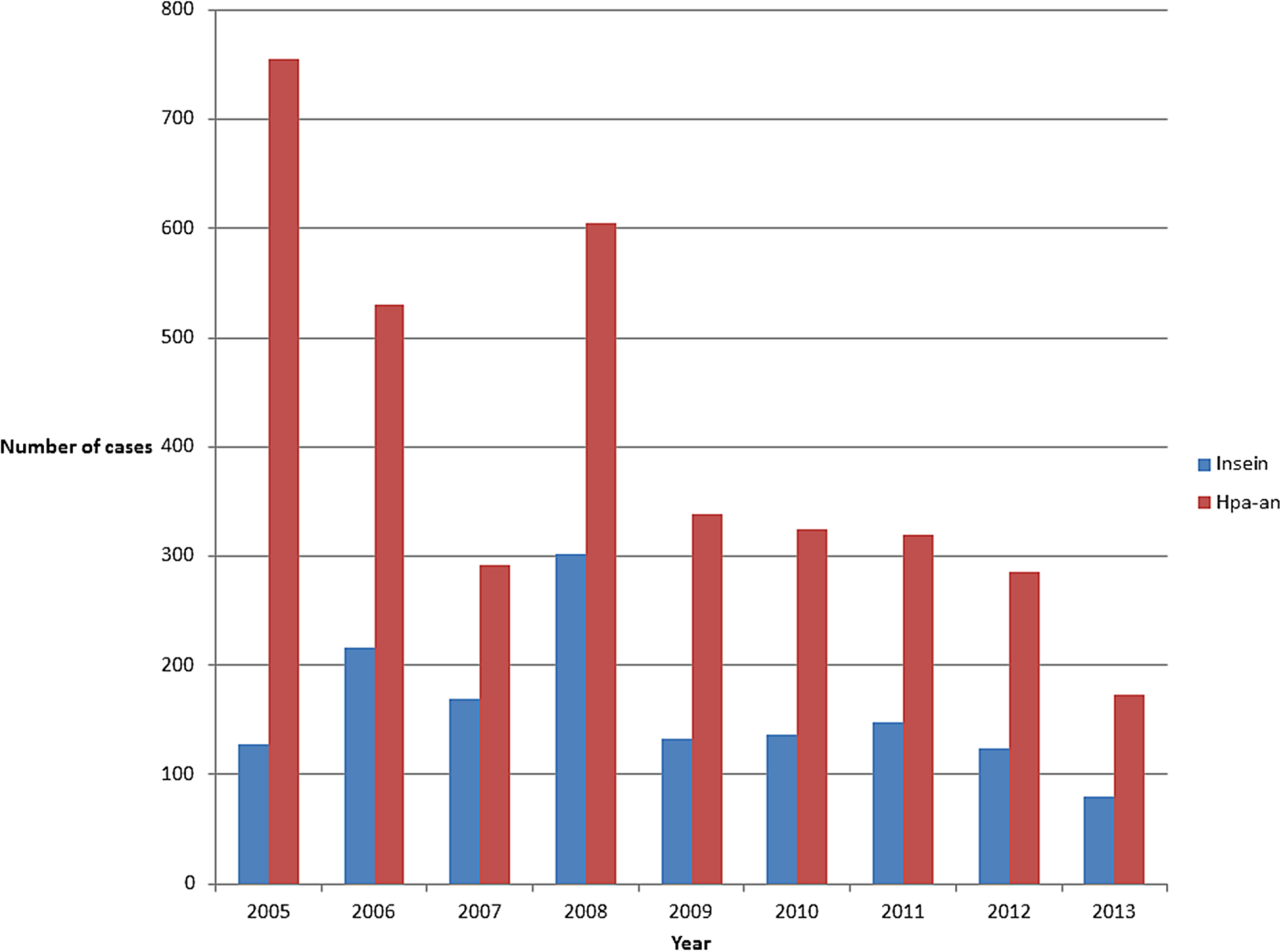
Incidence of malaria hospitalization at the two study sites since 2005*. * Includes all patients admitted to the hospital while the audit documents only adults admitted to the medical ward. Data source: personal correspondence Dr Thet Thet Mu, Deputy Director, Department of Health Planning, Ministry of Health, Myanmar.

From 1 January to 31 December, 2013, 90 patients were admitted to hospital with malaria at the two study sites; 56 at Hpa-an Hospital and 34 at Insein Hospital; this represented a continued decline in malaria hospitalization at both sites (Figure 1). There were few differences between patients at the study sites (Table 2), although patients admitted to Insein Hospital were more likely to have a history of travel in the previous four weeks (82 *versus* 11%, p = 0.0001). No patient had a RCAM score documented in the medical record, but all patients had a GCS and respiratory rate recorded on admission permitting its retrospective determination.

**Table 2:** **Characteristics of the cases admitted to the adult medical ward**

Variable*	**Total**	**Insein**	**Hpa-an**	**p^**
Number of cases	90	34	56	
Male sex	63 (70%)	29 (85%)	34 (61%)	0.02
Age (years)	34 (21–42)	31 (19–37)	35 (23–51)	0.17
Significant co-morbidities	20 (22%)	11 (32%)	9 (16%)	0.07
Duration of admission (days)	6 (4–8)	6 (4–8)	6 (4–7)	0.31
Died	7 (8%)	3 (9%)	4 (7%)	1
*P. falciparum* mono-infection	62 (69%)	24 (71%)	38 (68%)	0.79
*P. vivax* mono-infection	11 (12%)	5 (15%)	6 (11%)	0.57
Mixed infection	17 (19%)	5 (15%)	12 (21%)	0.43
Number of WHO severity criteria	2 (1–3)	2 (1–3)	2 (1–2)	0.69
RCAM score	1(1–1)	1 (1–2)	1(1–1)	0.48
History of travel in prior 4 weeks	34 (38%)	28 (82%)	6 (11%)	<0.001

*All numbers represent the raw number (%) or the median (interquartile range).

^Statistical difference between the two study sites.

**Figure 2:**
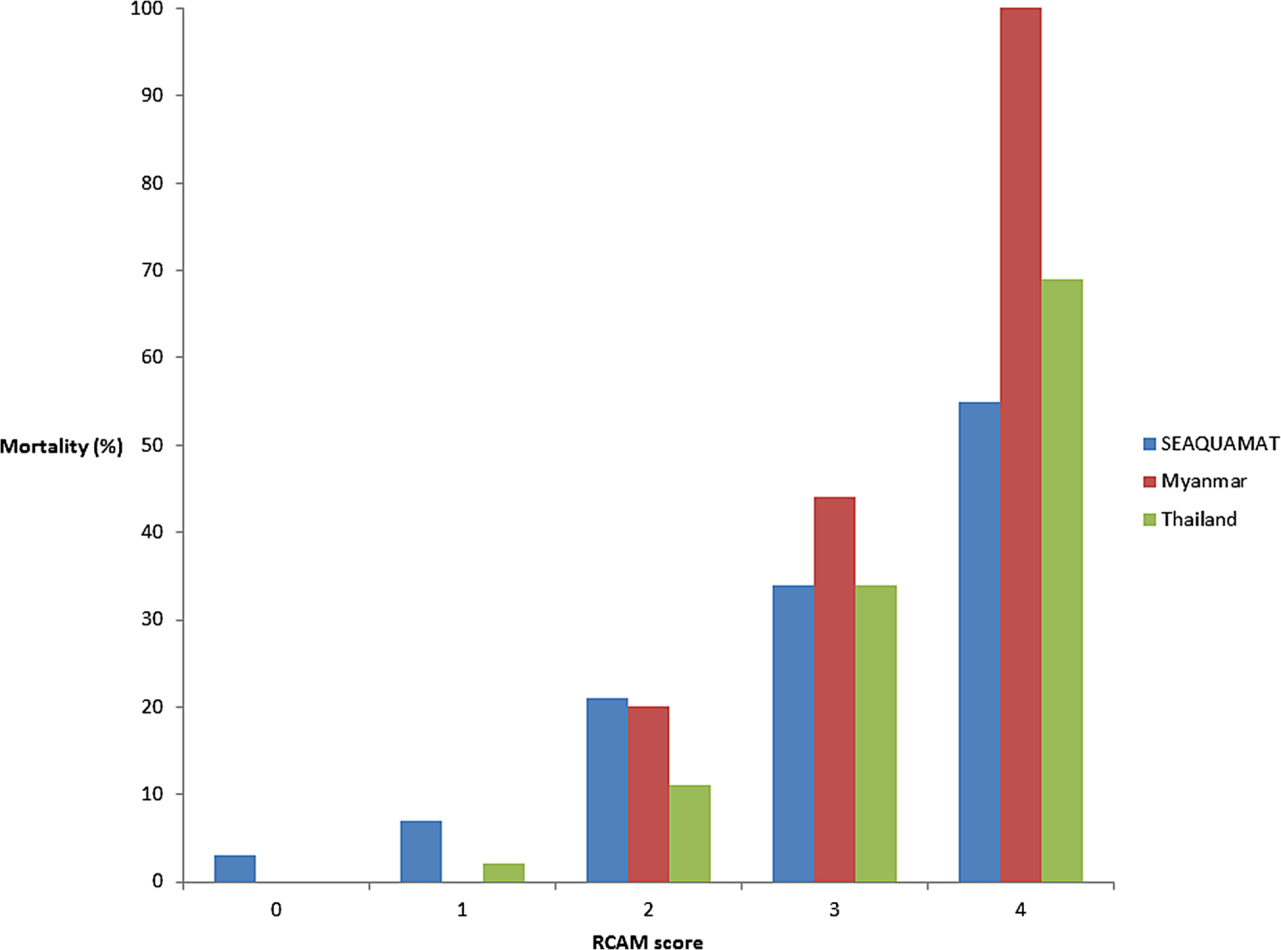
**Ability of the RCAM score to predict outcome in this and previous series [** 11 **,** 14 **].**

**Figure 3:**
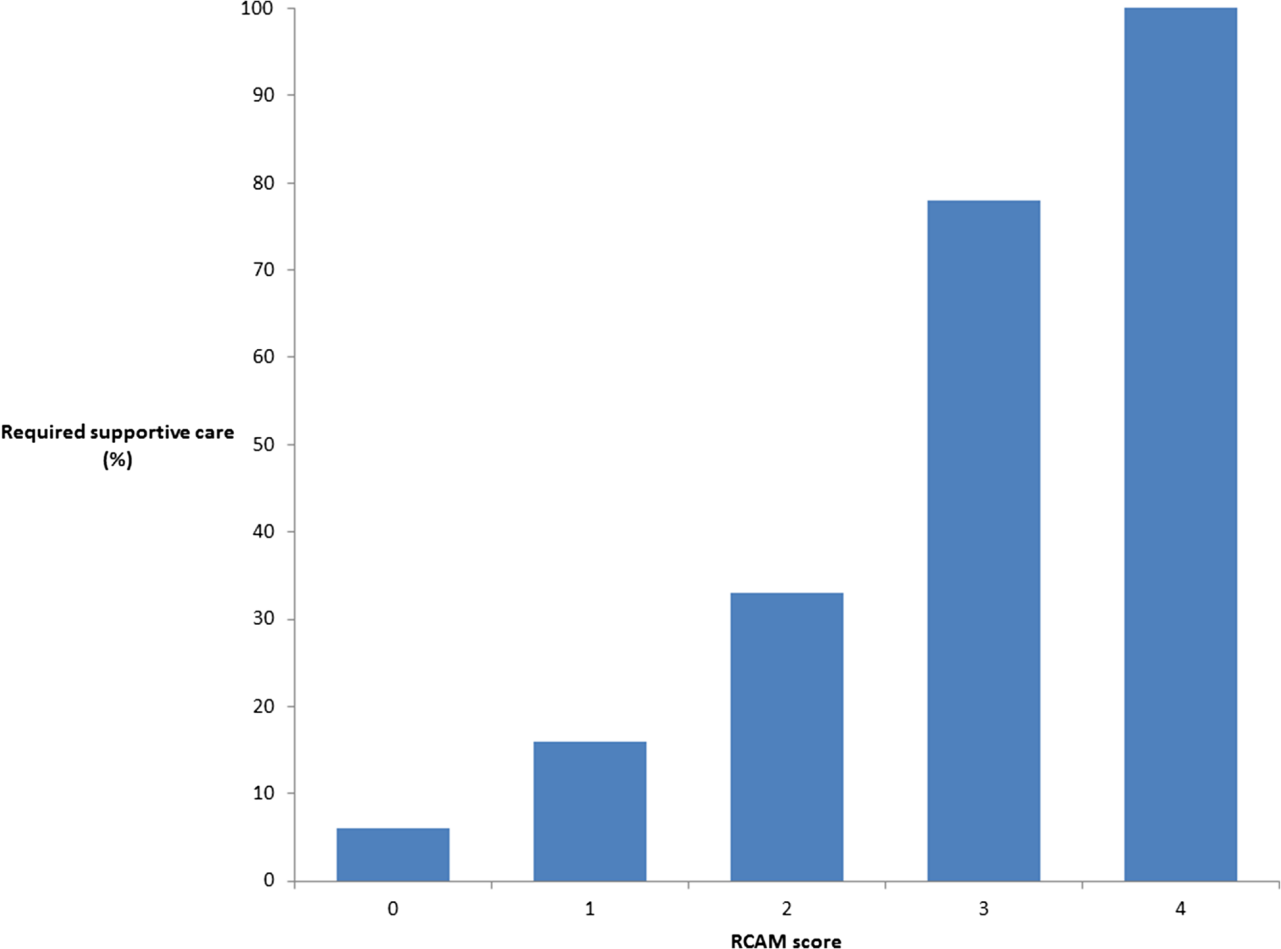
**Ability of the RCAM score to predict a requirement for supportive care.**

Overall 7/90 (8%) of patients in the series died; deaths tended to occur early in patients’ hospitalization with 5/7 (71%) occurring within the first 24 hours. All deaths occurred in patients infected with *Plasmodium falciparum*. The median (IQR) age of the patients that died was 32 (24–39) years; only 3/7 (43%) had co-morbidities (HIV infection, alcoholism and chronic anaemia, respectively). No patients with a RCAM score <2 on admission died (positive predictive value for survival 100% (95% confidence interval: 94.9-100) and the case-fatality rate increased steadily with rising RCAM score (p = 0.0001) (Figure 2, Table 3). A patient’s RCAM score on admission was also predictive of a requirement for supportive therapy: 9/70 (13%) of patients with a RCAM score <2 on admission required a blood transfusion, vasopressor support or oxygen supplementation during their hospitalization compared with 12/20 (60%) of patients with a RCAM score ≥2 (p < 0.0001) (Figure 3). A patient’s admission oxygen saturation (determined with pulse oximetry) was also a strong predictor of death before discharge (Table 4). Patients with an admission oxygen saturation below 96% on room air were far more likely to die before discharge (odds ratio 17.3 (95% confidence interval 2.9-101.2, p = 0.002); 5/7 (71%) deaths had an oxygen saturation below this level. The patients that died satisfied a median (IQR) of 4 (3–6) documented WHO severity criteria on admission compared with 2 (1–2) in survivors (p = 0.0002). The only WHO criteria that were significantly associated with outcome in this series were an elevated respiratory rate (>30 breaths per minute, p = 0.009), impaired GCS (GCS <15, p < 0.001) and hyperparasitaemia (≥3+ parasites on semi-quantitative counting, p = 0.03). The fact that some of the WHO criteria lacked an association with mortality was, at least partly, the result of incomplete data: haemoglobin and plasma creatinine were not commonly documented and plasma bicarbonate and lactate were not measured at either site. Other severity criteria occurred infrequently: shock (systolic blood pressure < 80 mmHg with cool peripheries) was present in only 3 cases, 2 of which survived.

**Table 3:** **RCAM score on admission and association with outcome**

**RCAM score**	**Survivors**	**Deaths**	**All**
0	16	0	16
1	55 (98.2%)	0	55
2	8 (80%)	2 (20%)	10
3	5 (56%)	4 (44%)	9
4	0	1 (100%)	1

**Table 4:** **Prognostic utility of the vital signs in the cases admitted to the adult medical ward in 2013**

**Variable**	**Survivors (83)**	**Deaths (7)**	**p**
Temperature (°C)	38 (37.2-39.1)	38 (36.6-39.6)	0.52
Heart rate (beats per minute)	100 (88–108)	120 (92–128)	0.04
Mean arterial pressure (mmHg)	83.2 (76.5-93.2)	76.6 (59.9-89.8)	0.2
Respiratory rate (breaths per minute)	22 (20–24)	26 (24–36)	0.002
Glasgow Coma Scale	15 (15–15)	9 (8–13)	0.0001
Oxygen saturation (%)	97 (96–98)	93 (87–96)	0.003

All numbers represent the median (interquartile range).

The majority of patients had *P. falciparum* mono-infection: 62/90 (69%) *versus* 17/90 (19%) with mixed infection and 11/90 (12%) with vivax mono-infection. Patients with *Plasmodium vivax* mono-infection generally had less severe disease; all 11 patients had prostration requiring hospitalization; three (27%) were also jaundiced but these patients all lacked any other organ dysfunction; one had a history of dark stools suggesting gastrointestinal bleeding, but there was no bleeding witnessed after hospitalization. No patients with *P. vivax* mono-infection had impaired consciousness compared with 20/79 (27%) of patients with *P. falciparum* infection (p = 0.11). Acute kidney injury (AKI) was not suspected clinically in any of the patients with *P. vivax* mono-infection and the plasma creatinine was normal (range: 62–88 μmol/L) in all five who had a value documented. By comparison, AKI (plasma creatinine >176 μmol/L) was present in 8/28 (29%) of patients with *P. falciparum* infection with a plasma creatinine recorded. Anaemia (haemoglobin concentration <11.5 g/dL) was present in 18/20 (90%) of patients with *P. falciparum* infection who had a haemoglobin concentration documented; the two patients with P. vivax mono-infection and a documented haemoglobin were not anaemic. No patient with *P. vivax* mono-infection required supportive care (blood transfusion, supplemental oxygen or vasopressor support) during their admission. By contrast supportive care was required in 21/79 (27%) of patients infected with *P. falciparum* (p = 0.06) (Table 5). The median (IQR) duration of hospitalization in patients with *P. vivax* mono-infection was 4 (4–5) days *versus* 6 (4–8) days in patients infected with *P. falciparum,* p = 0.02). All patients with *P. vivax* mono-infection had a RCAM score <2.

**Table 5:** **Comparison of cases with** ***Plasmodium vivax*** **mono-infection with** ***Plasmodium falciparum*** **infection**

	**n**	***P. falciparum*** **(79 cases)**	***P. vivax*** **(11 cases)**	**p**
Mortality	90	7 (9%)	0	0.59
Duration of hospitalization* (days)	90	6 (4–8)	4 (4–5)	0.02
Temperature (°C)	90	38 (37.2-39.1)	38.5 (36.9 -38.5)	0.67
Heart rate (beats per minute)	90	100 (88–110)	104 (78–120)	0.93
Mean arterial pressure (mmHg)	90	83.2 (76.5-93.2)	79.9 (76.5-83.2)	0.45
Respiratory rate (breaths per minute)	90	22 (20–24)	22 (20–24)	0.47
Glasgow Coma Scale score	90	15 (14–15)	All cases had GCS 15	0.06
Oxygen saturation (%)	86	97 (96–98)	98 (98–98)	0.01
Plasma creatinine (μmol/L)	33	92 (64–200)	64 (63–80)	0.16
Haemoglobin (g/dL)	22	7.8 (6.7-9.9)	12.2 (11.7 – 12.6)	0.07
Required blood transfusion number (%)	90	13 (17%)	0	0.16
Required supplemental O_2_ number (%)	90	8 (10%)	0	0.59
Required vasopressor support number (%)	90	6 (8%)	0	0.44
Acute kidney injury ^ number (%)	33	8 (10%)	0	0.59
Spontaneous bleeding number (%)	90	12 (15%)	0	0.35

All numbers represent the raw number (%) or the median (interquartile range).

*Only includes survivors.

^Creatinine documented > 176 μmol/L.

## Discussion

Although this clinical audit was conducted at major regional centres in Lower Myanmar, limited diagnostic facilities at both sites precluded the application of many of the traditional criteria used to identify patients with severe malaria. However a few simple clinical indices promptly identified the patients who were at greater risk of a complicated course. Patients’ RCAM score on admission was strongly linked to outcome. Not only did a RCAM score ≥2 identify all the patients that would later die, it also strongly predicted a requirement for supportive care, potentially allowing triage of these patients to areas in the hospital where closer monitoring is possible. The mortality rates with each increment of the RCAM score in patients receiving everyday care were quantitatively very similar to those seen in the studies of patients enrolled in clinical trials [11,12], suggesting generalizability of the score.

As in previous multi-centre studies of adults with severe *P. falciparum* infection, oxygen saturation determined on admission with pulse oximetry was also a powerful prognostic indicator [12]. Pulse oximetry has been used to measure arterial oxygen saturation non-invasively for over 30 years [15]. It is used so widely in everyday medical practice that it is sometimes referred to as the ‘fifth vital sign’ [16]. Although *Plasmodium* infection can lead directly to acute pulmonary oedema, pulse oximetry has the advantage of also identifying respiratory co-morbidity, aspiration pneumonia or concurrent community-acquired or nosocomial pneumonia, all of which are associated with high mortality [17-19]. Even a small drop in oxygen saturation in patients with falciparum malaria is significant as the metabolic acidosis that is frequently present leads to an increased minute ventilation [18]. In a previous multi-centre study, patients with saturation of ≤95% were more than twice as likely to die before discharge [12]; in this smaller series the odds ratio for death was even greater. These data support a more prominent role for pulse oximetry in disease management algorithms.

These three clinical signs (GCS, respiratory rate and pulse oximetry) can be measured by even junior health care workers. An advantage of management algorithms using simple clinical signs is that respiratory rate, pulse oximetry are routinely collected in all hospitalized patients, while GCS is usually recorded in all those with impaired consciousness. This allows integration of patient care, avoiding complex individual disease care pathways [20]. This is particularly important as malaria incidence declines; a derangement in the vital signs is of equal relevance in patients with severe malaria, pre-eclampsia, ischaemic heart disease or a head injury. Early recognition of a change in the vital signs is widely used in the industrialized world to promptly identify at-risk patients and has been shown to improve outcomes [21-23].

Although it has been traditionally thought that *P. vivax* infection is much less likely to progress to severe malaria than *P. falciparum* infection, the notion that *P. vivax* is a ‘benign’ infection has been vigorously challenged [24,25] and in the last decade there has been a marked increase in reports of severe *P. vivax* infection [26]. Coma, renal failure, multi-organ failure and death have all been described [27,28], although there remains uncertainty about the contribution of co-morbidities to these clinical presentations [26] and few reports have provided population denominators to estimate their incidence [29]. It is unclear to what extent this increased documentation is the result of the emergence of virulent strains, previously underestimated severity, or a renewed interest in the parasite [30,31]. In this series hospitalization with *P. vivax* mono-infection was not frequent and no case had clinical manifestations beyond prostration and jaundice; there was no evidence of the more severe clinical phenotypes described in other series. It is notable that all the patients with *P. vivax* mono-infection had RCAM scores <2 and all survived without blood transfusion, vasopressor or oxygen support. These findings are consistent with the rarity of reports of severe disease in patients with *P. vivax* mono-infection from other countries in the Greater Mekong region [1].

The decreasing incidence of malaria requiring hospitalization reported in this study parallels the findings of many centres globally and reflects the success of the Roll Back Malaria Partnership [32,33]. However Myanmar still bears a significant – and disproportionate – burden of disease. In 2012 Myanmar reported 480,000 malaria cases and while the country is home to less than 3% of Southeast Asia’s population, it is responsible for 49% of the region’s reported malaria deaths [32]; Myanmar is also the country most imminently threatened by the evolving resistance to artemisinin therapy [34]. It is likely that clinicians in Myanmar will be caring for patients with malaria for years to come. While the vast majority of cases of malaria will be successfully managed on an outpatient basis, even young and otherwise healthy individuals can deteriorate rapidly: all but one of the patients that died in this series was younger than 40, over half had no co-morbidities. While improved access to ICU level care including RRT and mechanical ventilation will improve outcomes [35], and strategies are in place in an attempt to increase access to this care in Myanmar [36], the use of simple inexpensive clinical management pathways is likely to improve outcomes in the short term [37].

The study had several major limitations. The audit was retrospective and accordingly data collection was incomplete; limited documentation of some laboratory data in particular precluded detailed evaluation of its utility in patient assessment. However, the fact that the data required for derivation of the RCAM score were documented in all patients’ records illustrates its potential applicability in routine practice. Molecular data were not available to confirm the species of the infecting malaria parasite(s), however the data collected here – presumptive speciation with RDT, confirmed by an experienced microscopist – represents real world practice. Although clinical indices appeared useful in predicting patients’ in-hospital course, this does not obviate the need for a strict and specific case definition which includes laboratory indices; such a definition is important for clinical researchers, particularly those studying malaria pathophysiology.

There are also several caveats: although clinical indices appeared helpful in patient triage this series, laboratory and radiological investigations remain essential to confirm clinical impressions; the study’s findings in no way suggest that diagnostic laboratory testing can be routinely deferred. Analysis was performed using only patient data recorded on admission; even if a patient is triaged as low-risk this does not preclude the possibility of complications developing during their hospitalization, although this did not transpire in this series. The data were collected in adults in referral hospitals, and do not necessarily reflect the utility of clinical signs in children or in other settings. Finally, as in the management of patients with any illness, the use of prediction tools should not override a physician’s clinical judgement.

## Conclusions

Malaria incidence is declining in Myanmar, however patients continue to die from falciparum malaria. Early identification of patients at greatest risk of a complicated course can facilitate resource allocation and may improve outcomes. As patients with malaria are almost universally managed in the resource-poor setting – frequently by junior health care workers – patient management algorithms should be simple, practical and integrated with the care of other patients. Triage of patients based on simple clinical indices appears to achieve these aims and appears generalizable outside the research setting, although prospective validation is required to confirm the safety, accuracy and efficacy of such an approach.

### Consent

Written informed consent was obtained from the patient for the publication of this report and any accompanying images.
